# Severe oligomeric tau toxicity can be reversed without long-term sequelae

**DOI:** 10.1093/brain/awaa445

**Published:** 2021-01-23

**Authors:** Alfonso Martinisi, Martin Flach, Frederik Sprenger, Stephan Frank, Markus Tolnay, David T Winkler

**Affiliations:** 1 Institute of Medical Genetics and Pathology, University Hospital Basel, CH-4031 Basel, Switzerland; 2 Department of Neurology, University Hospital Basel, CH-4031 Basel, Switzerland; 3 Neurology, Medical University Clinic, Kantonsspital Baselland, 4410 Liestal, Switzerland

**Keywords:** Alzheimer’s disease, neurodegeneration, tau, seeding, oligomers

## Abstract

Tau is a microtubule stabilizing protein that forms abnormal aggregates in many neurodegenerative disorders, including Alzheimer’s disease. We have previously shown that co-expression of fragmented and full-length tau in P301SxTAU62^*on*^ tau transgenic mice results in the formation of oligomeric tau species and causes severe paralysis. This paralysis is fully reversible once expression of the tau fragment is halted, even though P301S tau expression is maintained. Whereas various strategies to target tau aggregation have been developed, little is known about the long-term consequences of reverted tau toxicity. Therefore, we studied the long-term motor fitness of recovered, formerly paralysed P301SxTAU62^*on-off*^ mice. To assess the seeding competence of oligomeric toxic tau species, we also inoculated ALZ17 mice with brainstem homogenates from paralysed P301SxTAU62^*on*^ mice. Counter-intuitively, after recovery from paralysis due to oligomeric tau species expression, ageing P301SxTAU62^*on-off*^ mice did not develop more motor impairment or tau pathology when compared to heterozygous P301S tau transgenic littermates. Thus, toxic tau species causing extensive neuronal dysfunction can be cleared without inducing seeding effects. Moreover, these toxic tau species also lack long-term tau seeding effects upon intrahippocampal inoculation into ALZ17 mice. In conclusion, tau species can be neurotoxic in the absence of seeding-competent tau aggregates, and mice can clear these tau forms permanently without tau seeding or spreading effects. These observations suggest that early targeting of non-fibrillar tau species may represent a therapeutically effective intervention in tauopathies. On the other hand, the absent seeding competence of early toxic tau species also warrants caution when using seeding-based tests for preclinical tauopathy diagnostics.

## Teaser

Co-expression of fragmented and full-length tau in P301SxTAU62^*on*^ transgenic mice results in reversible tau oligomer-induced paralysis. Martinisi *et al.* show that tau oligomers can be cleared without provoking late tau seeding or neurodegeneration, suggesting that non-fibrillar tau may be a promising therapeutic target.

## Introduction

Tau is a soluble protein acting as a microtubule stabilizer in neuronal cells. Under pathological conditions it becomes hyperphosphorylated and eventually forms intracellular aggregates. Tau aggregates are a hallmark of Alzheimer’s disease and other neurodegenerative disorders, including forms of frontotemporal dementia (Spillantini and Goedert, 2013; Spires-Jones and Hyman, 2014). The mechanisms underlying pathological tau aggregation remain only partly understood. Tau is thought to aggregate through the formation of oligomeric species and subsequent aggregation into fibrils, culminating in the formation of tangles. Tau aggregation can spread towards anatomically connected regions in a prion-like manner (Clavaguera *et al.*, 2009; Goedert *et al.*, 2017).

We have recently shown that tau toxicity can be mediated by oligomeric tau species (Ozcelik *et al.*, 2016). Co-expression of full-length P301S mutant tau with a 3R tau_151-421_ fragment *(*Δtau_151-421_) in 3-week-old P301SxTAU62^*on*^ transgenic mice leads to the formation of soluble high molecular weight tau oligomers. In the P301SxTAU62^*on*^ mouse model, high molecular weight tau oligomers are sufficient to cause extensive nerve cell dysfunction and severe motor palsy, both of which occur in the absence of insoluble tau aggregates or neurofibrillary tangles (NFTs) (Ozcelik *et al.*, 2016). Strikingly, once the doxycycline-inducible expression of Δtau_151-421_ is switched off in these mice, their severe phenotype reverses within 3 weeks, and animals regain their motor competence, even though heterozygous P301S mutant tau expression is maintained (Ozcelik *et al.*, 2016). This phenotype reversibility renders P301SxTAU62^*on-off*^ mice suitable for investigating potential long-term consequences of reverted tau toxicity. The relevance of oligomeric tau toxicity is being increasingly recognized, and recent studies have shown that it can also be pharmacologically attenuated (Bittar *et al.*, 2019; Lo *et al.*, 2019; Abskharon *et al.*, 2020; Lo Cascio *et al.*, 2020; Puangmalai *et al.*, 2020). Hypothesized to depend on oligomer conformation, tau oligomeric toxicity can also be aggravated by biological interactions with chaperones (Oroz *et al.*, 2018), RNA-binding proteins (Jiang *et al.*, 2019), and nuclear complexes (Eftekharzadeh *et al.*, 2018). Because of their interaction with cellular components and their importance in tau propagation, tau oligomers have been considered crucial for tau-mediated neurodegeneration.

Long-term effects after recovery from oligomeric, non-fibrillar tau toxicity have not yet been assessed. Here, we study the late effects of reverted tau stress in aged P301SxTAU62^*on-off*^ mice. Seeding and spreading of tau pathology have been widely studied in human tauopathies as well as in tau transgenic mouse models (Maeda *et al.*, 2007; Clavaguera *et al.*, 2009; Sanders *et al.*, 2014; Mudher *et al.*, 2017). Based on this accumulated evidence, we hypothesized that the tau oligomers abundantly present in young paralysed P301SxTAU62^*on*^ mice might act as seeds for tau accumulation, as P301S full-length tau expression is maintained, while doxycycline-driven Δtau_151-421_ expression is switched off. To our surprise, P301SxTAU62^*on-off*^ mice that had recovered from neurotoxic stress and motor palsy did not develop worse motor function nor more pronounced tau pathology during ageing when compared to their P301S tau heterozygous littermates. This demonstrates that P301SxTAU62^*on-off*^ mice clear their toxic tau aggregates without seeding NFT formation, and hence do not show pronounced motor decline upon ageing.

To study whether the toxic tau species causing palsy in young P301SxTAU62^*on*^ mice also lack classical seeding competence, we next stereotactically inoculated brainstem homogenates of paralysed P301SxTAU62^*on*^ into ALZ17 human wild-type tau transgenic mice. In contrast to ALZ17 mice seeded with P301S brainstem homogenates containing fibrillary tau species (Clavaguera *et al.*, 2009), ALZ17 mice inoculated with P301SxTAU62^*on*^ homogenate did not develop fibrillar tau pathology, arguing against a seeding competence of the early toxic tau forms in P301SxTAU62^*on*^ mice.

Lacking seeding competence of non-fibrillar toxic tau species as well as absent long-term sequelae after halting their expression warrants the exploration of early therapeutic interventions targeting pre-fibrillar tau species. Our observations may also question the reliability of seeding-based assays for early preclinical tauopathy diagnostics.

## Materials and methods

### Mice

Transgenic homozygous mice expressing human ALZ17 mutant tau (ALZ17 mice) (Probst *et al.*, 2000), transgenic homozygous mice expressing human P301S mutant tau (P301S mice) (Allen *et al.*, 2002), transgenic heterozygous mice expressing human Δtau_151-421_ (TAU62 mice) (Ozcelik *et al.*, 2016), and non-transgenic C57BL/6J (BL6 mice) control mice were used. P301S and TAU62 mice were interbred to obtain double transgenic P301SxTAU62 mice (Ozcelik *et al.*, 2016). All animal experiments were performed in compliance with protocols approved by the Committee for Animal Care and Animal Use of the Canton of Basel.

### Behavioural assessments

Motor behaviour, including gait ataxia, tremor, and hindlimb reflexes was assessed. Quantitative motor testing was performed by the grid test in which mice were placed on a vertical mesh grid and the latency to fall off the grid was recorded for 3 min. Motor coordination and balance were assessed using the Panlab Harvard Rotarod (Harvard Apparatus). The rotarod starts at a speed of 4 rpm and accelerates by 1 rpm every 3 s. In both the grid test and the rotarod assay, mice were tested for three consecutive days with three trials per day, with minimum rest intervals of 5 min, and the mean latency to fall was documented. Results were obtained by averaging the daily means of three consecutive days.

### Stereotactic surgery

Three-month-old ALZ17 mice were anaesthetized with a mixture of ketamine and xylazine and placed on a heating pad to maintain body temperature. Mice were stereotactically injected into the right hippocampus (A/P: −2.5 mm from bregma; L: −2.0 mm; D/V: −1.8 mm) using a Hamilton syringe. Brainstem homogenates of 6-month-old paralysed homozygous P301S mice and 3-week-old paralysed P301SxTAU62^*on*^ mice were prepared for inoculation in ALZ17 mice. Brainstems were weighed and diluted 1:10 in phosphate-buffered saline (PBS) for seeding. After dilution, samples were homogenized using an Ultraturrax T8 (IKA Labortechnik) and sonicated briefly (Bandelin SONOPULS; 90% power, 10% cycle, 10-s pulses). Homogenates were then centrifuged at 4000*g* for 20 min at 4°C, and aliquots of the supernatant were stored at −70°C for later usage. During inoculation, each mouse received 5 μl of brainstem homogenate at a speed of 1.25 μl/min. Following injection, the needle was kept in place for an additional 3 min before withdrawal. The surgical area was cleaned with sterile saline and the incision sutured. Mice were monitored until recovery from anaesthesia, post-interventional analgesia was administered, and animals were checked regularly following surgery. After 20 months of incubation, the seeded mice were sacrificed.

### Immunohistochemistry

Mice were deeply anaesthetized and transcardially perfused with 20 ml cold PBS, followed by 20 ml 4% paraformaldehyde in PBS. The brains were dissected and post-fixed overnight. Following paraffin embedding, 4-μm coronal sections were cut from the brains of seeded mice, whereas 4-μm sagittal sections were prepared from the brains of mice used for the behavioural tests. Sections were silver-impregnated following the method of Gallyas-Braak to visualize filamentous tau pathology. Haematoxylin and eosin staining was performed for morphological analysis. For immunohistochemistry, the following anti-tau antibodies were used: AT8 (1:1000, Pierce Biotechnology), AT100 (1:1000, Pierce Biotechnology), and TauC3 (1:1000, Santa Cruz Biotechnology). Secondary antibodies were from Vector Laboratories (Vectastain ABC kit). Magnified pictures (×10, ×20 and ×40) of the stained slides were taken with an Olympus BX43 Upright Microscope (Life Sciences Solutions).

### Western blots

For the western blots comparing total and soluble tau in 16-month-old P301S heterozygous (P301Shet) and P301SxTAU62^*on-off*^ mice, brains were homogenized in cold extraction buffer 20% (w/v) [25 mM Tris-HCl pH 7.4, 150 mM NaCl, 1 mM EDTA, 1 mM EGTA, 5 mM Na pyrophosphate, 10 mM B-glycerophosphate, 30 mM NaF, 10 mM Na vanadate, with addition of 100 μl/10 ml PMSF (0.1 M) just before use, and one Pierce protease and phosphatase inhibitor mini tablet, EDTA-free (Pierce Biotechnology)] using a Polytron Homogenizer (Thomas Scientific). An aliquot of the resulting homogenate was collected as total tau. Samples were subsequently centrifuged at 80 000*g* for 15 min using an ultracentrifuge (Beckman Coulter; OptimaTM L-70K Ultracentrifuge), and an aliquot of the supernatant was collected as soluble tau. For the western blot comparing total tau in 3-month-old mice, one-half of the mouse brain was dissected into forebrain and brainstem, and frozen in liquid nitrogen or on dry ice. Brainstems were weighed and diluted 1:10 in TBS-Complete. Subsequently, samples were homogenized using an Ultraturrax T8 (IKA Labortechnik) and briefly sonicated (Bandelin SONOPULS; 90% power, 10% cycle, 10 s pulses). Homogenates were then centrifuged at 4000*g* for 20 min at 4°C, and aliquots of the supernatant stored at −70°C for later usage. Western blots were then carried out under non-reducing conditions using appropriate amounts of protein, 4× NuPAGE^®^ LDS sample buffer, and deionized water. NuPAGE^®^ (10×) reducing agent was used to obtain reducing conditions. Following appropriate preparation, samples were loaded onto a 7% NuPAGE^®^ Tris-acetate gel. After the removal of gels from the cassette and activation of PVDF membrane (Amersham Biosciences) samples were transferred on the PVDF membrane using the XCell IITM Blot Module. Unspecific binding epitopes were blocked with 5% non-fat milk in PBS-Tween, followed by incubation with primary antibody overnight at 4°C on a shaker. After washing with PBS-Tween, the membrane was incubated with horseradish peroxidase (HRP)-conjugated anti-mouse or -rabbit secondary antibody at room temperature. The membrane was then washed again in PBS-Tween and detected by electrochemiluminescence (ECL) (GE Healthcare). The anti-tau antibody used for the western blots was HT7 (1:1000, Pierce Biotechnology).

### Sarkosyl extraction and immunoelectron microscopy

Following PBS perfusion, mouse brainstem tissue was dissected and frozen in liquid nitrogen. Sarkosyl extraction was performed as previously described (Delobel *et al*., 2008). Briefly, the brainstem tissue was homogenized in A68 buffer (0.5 ml of 800 mM NaCl, 10% sucrose, 10 mM Tris-HCl pH 7.4, 1 mM EGTA) using a Kinetica polytron. Samples were centrifuged at 5000*g* for 15 min. The collected supernatant was analysed as total tau samples. Following sarkosyl addition to 1% and shaking for 1 h, samples were centrifuged at 80 000*g* for 30 min. The resulting pellet was resuspended in 50 mM Tris-HCl pH 7.4.

For immunoelectron microscopy, aliquots were placed on carbon-coated 400 mesh grids and allowed to dry partially. Grids were blocked in droplets of 0.1% gelatin (Sigma G7041, Sigma-Aldrich) and stained with HT7 primary antibody (1:50; Pierce). Grids were then washed briefly with blocking buffer, stained with anti-mouse IgG-Gold secondary antibody (Sigma G7652), washed with water, and stained with 2% uranyl acetate. Electron microscopy was performed using a FEI Tecnai Spirit TEM at a magnification of ×21 000 and images recorded using a Gatan Orius SC200B CCD camera (Gatan).

### Dot blots

For dot blots, serum of 3-month-old P301Shet and P301SxTAU62^*on-off*^ mice was separated from the clot by centrifuging the samples at 1000 rpm for 15 min at 4°C, with the remaining supernatant aliquoted and stored at −20°C, for later usage. A nitrocellulose membrane (Bio-Rad Laboratories, Inc) was then divided in a grid to allow later incubations with 3-month-old P301Shet and P301SxTAU62^*on-off*^ sera and HT7 antibody as positive control at 1:1000 dilutions. 2N4R wild-type tau monomers (0.2 mg/ml) were applied to a 4 × 3 grid. Unspecific binding epitopes were blocked with 5% bovine serum albumin (BSA) in PBS-Tween, followed by a 30-min long incubation at room temperature with previously extracted sera from the mice and HT7 antibody as positive control. After washing with PBS-Tween, the membrane was incubated with HRP-conjugated anti-mouse secondary antibody at room temperature, washed again in PBS-Tween, and detected by ECL (GE Healthcare).

### Statistical analysis

To evaluate behavioural assessments, one-way ANOVA followed by *post hoc* Student’s *t*-tests and Bonferroni correction for multiple comparisons were applied. *P*-values < 0.05 were considered significant. To estimate soluble tau and total tau expression from western blots of 3- and 16-month-old mice, the protein bands were normalized to GAPDH protein standard, and quantified using ImageJ software; generated mean ratio values were compared by Student’s *t*-test. To determine the effect of early neurotoxic stress in aged mice, AT8 and Gallyas-positive neurons were semiquantitatively assessed in brainstem regions. Three sections per animal were analysed. The total area analysed per animal was comparable between animals (AT8: *P*-value = 0.34, Gallyas: *P*-value = 0.91). *P*-values calculated by Student’s *t*-tests were interpreted exploratory and not adjusted for multiple comparisons; *P*-values < 0.05 were considered significant. Box plots for Figs 3–6 were generated with R software. The lower and upper hinges correspond to the first and third quartiles (25th and 75th percentiles). The upper whisker extends from the hinge to the largest value, no further than 1.5 times IQR from the hinge (where IQR is the interquartile range, or distance between the first and third quartiles). The lower whisker extends from the hinge to the smallest value at most 1.5 times IQR of the hinge. Data beyond the end of the whiskers are called ‘outlying’ points and are plotted individually.

### Data availability

The datasets used and/or analysed during the current study are available from the corresponding author on reasonable request.

## Results

### Tau fragment induced severe reversible neurotoxicity in P301SxTAU62*^on^* mice in the absence of tau fibrils

**Figure 1:**
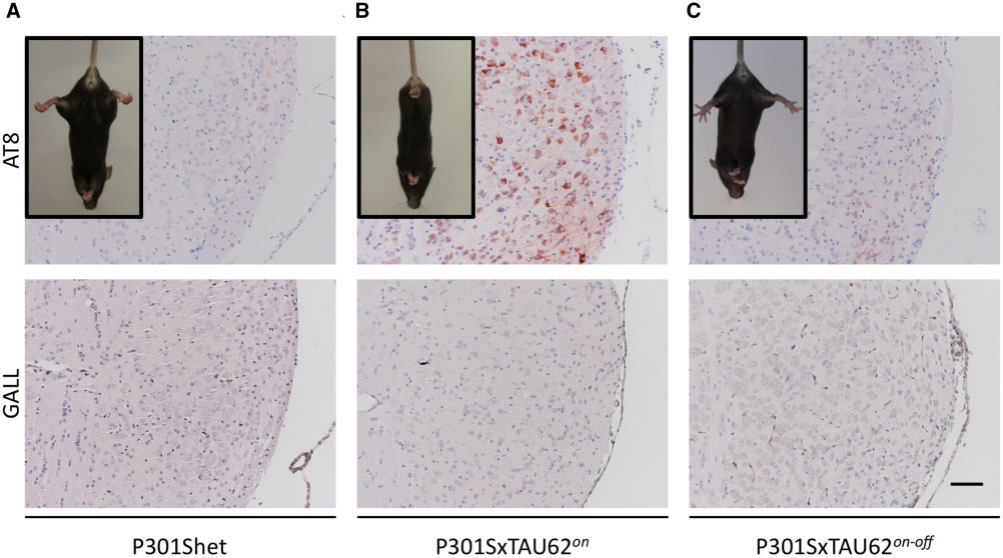
**Early neurotoxic stress depends on full-length and Δtau co-expression and is reversible.** (**A**) P301S heterozygous mice (P301Shet; *n *=* *5) at 21 days of age show no signs of paralysis upon tail suspension, and histological tests with AT8 antibody did not detect hyperphosphorylated tau or Gallyas-positive tau fibrils in the tegmental reticular nucleus. (**B**) Hindlimbs of P301SxTAU62^*on*^ mice (*n *=* *5) the same age as their heterozygous littermates are paralysed; upon histological characterization, paralysed P301SxTAU62^*on*^ mice show hyperphosphorylated tau but no Gallyas-positive tau fibrils in the same region. (**C**) At 3 weeks after halting doxycycline administration, P301SxTAU62^*on-off*^ mice (*n *=* *5) show recovered motor functions and reverted tau hyperphosphorylation. Scale bar = 75 µm (**A**–**C**).

P301SxTAU62^*on*^ mice co-expressing human Δtau_151-421_ with full-length P301S mutant tau were obtained by interbreeding P301S mice with TAU62 mice, where Δtau_151-421_ expression is regulated by a doxycycline-responsive promoter element (Ozcelik *et al.*, 2016). Three-week-old heterozygous P301S transgenic littermates (P301Shet) did not exhibit signs of motor dysfunction, as also illustrated by their normal tail suspension test (Fig. 1A). In contrast, P301SxTAU62^*on*^ mice of the same age showed a severe hindlimb palsy, only being able to move by the use of their forelimbs (Fig. 1B). Upon halting doxycycline administration to stop Δtau_151-421_ expression, this phenotype was fully reversible within 3 weeks, even though P301S tau expression continued (Fig. 1C).

Histological examination did not reveal any tau pathology in young P301Shet mice (Fig. 1A). In contrast, extensive AT8-positive pretangle stage tau pathology was found in paralysed, 3-week-old P301SxTAU62^*on*^ mice (Fig. 1B). Remarkably, 3 weeks after Δtau_151-421_ expression had been stopped, AT8-positive tau pathology was no longer detectable, and by the age of 6 weeks, P301SxTAU62^*on-off*^ mice had regained normal walking capability (Fig. 1C). Gallyas-Braak silver stain positive tau pathology was absent in 3-week-old P301Shet mice (Fig. 1A), their paralysed P301SxTAU62*^on^* littermates (Fig. 1B), as well as in recovered P301SxTAU62^*on-off*^ mice (Fig. 1C, for high magnification pictures see Supplementary Fig. 1).

**Figure 2:**
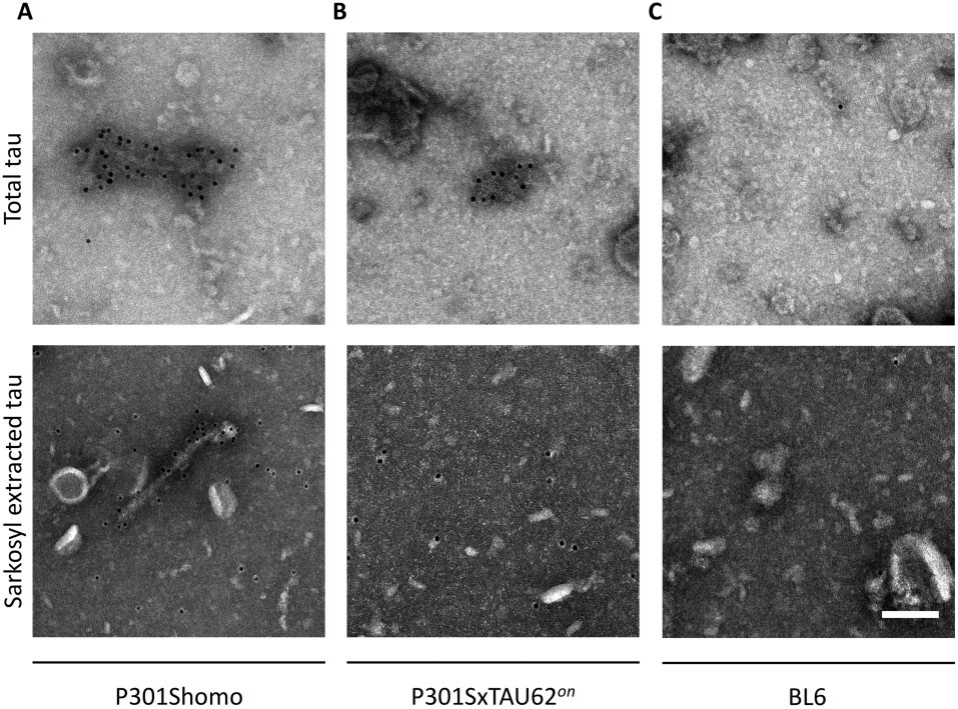
**Absence of tau filaments in paralysed P301SxTAU62^*on*^ mice.** (**A**) Immunoelectron microscopy shows HT7-positive tau filaments in total tau and sarkosyl extracts of brainstem tissue from a 6-month-old homozygous P301S mouse. (**B**) Total tau extract collected from a 3-week-old paralysed P301SxTAU62^*on*^ mouse reveals non-filamentous, oligomeric HT7 antibody positive tau structures while tau filaments are absent in its sarkosyl extract. (**C**) Absence of tau aggregates in a 3-week-old BL6 control mouse. The findings confirm that the severe neurotoxicity in P301SxTAU62^*on*^ mice does not depend on filamentous tau pathology, but is linked to hyperphosphorylated tau oligomers. Scale bar = 100 nm (**A**–**C**).

Immunoelectron microscopy confirmed the absence of tau filaments in brainstem samples of paralysed P301SxTAU62*^on^* mice (Fig. 2). Total tau, as well as sarkosyl extracts of brainstem homogenates collected from 6-month-old homozygous P301S tau transgenic mice, showed HT7-positive tau filaments (Fig. 2A). In contrast, only oligomeric, non-fibrillar tau structures were detectable in total tau extracts of 3-week-old paralysed P301SxTAU62^*on*^ mice, and no filamentous aggregated tau structures could be found in sarkosyl extracts from paralysed P301SxTAU62^*on*^ mice (Fig. 2B). Extracts from non-transgenic 3-week-old BL6 mice were used as negative controls (Fig. 2C). These observations demonstrate that the severe, but reversible motor palsy in P301SxTAU62^*on-off*^ mice is mediated by non-fibrillar tau species (Ozcelik *et al.*, 2016). A graphical representation of these findings is given in Supplementary Fig. 2.

### Absence of excessive motor impairment in aged P301SxTAU62^*on-off*^ mice after recovery from early palsy

To study the long-term effects of the severe non-fibrillar tau stress in P301SxTAU62^*on*^ mice, we followed their motor capabilities following initial recovery. As only Δtau_151-421_ expression is under the control of a doxycycline-responsive promoter element, recovered P301SxTAU62^*on-off*^ mice maintain expression of human P301S mutant full-length tau.

**Figure 3:**
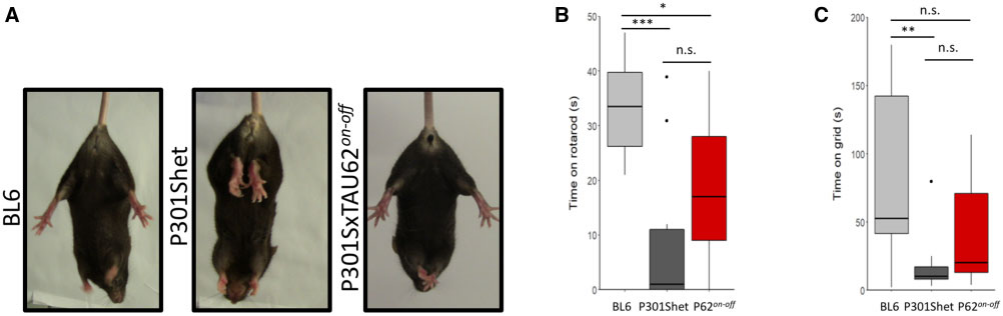
**P301SxTAU62^*on-off*^ mice do not develop an impaired motor phenotype upon ageing.** (**A**) Tail suspension revealed normal hindlimb spreading for BL6 mice, and pathological hindlimb spreading predominantly in heterozygous P301S transgenic littermates at 16 months of age, while this was less pronounced in age-matched P301SxTAU62^*on-off*^ (abbreviated P62^*on-off*^) mice. (**B**) Rotarod test comparing BL6 (*n *=* *10), P301Shet (*n *=* *15), and P301SxTAU62^*on-off*^ mice (*n *=* *13) at 16 months of age revealed that P301SxTAU62^*on-off*^ animals were not significantly more impaired than P301Shet mice that did not undergo early neurotoxic stress (*P*-value = 0.14). (**C**) Grid climbing of BL6 (*n *=* *10), P301Shet (*n *=* *17), and P301SxTAU62^*on-off*^ mice (*n *=* *11) at 16 months also showed that P301SxTAU62^*on-off*^ mice were not experiencing heavier motor impairment when compared to their heterozygous littermates (*P*-value = 0.09). n.s. = *P *>* *0.05; *P <* *0.05; **P <* *0.01; ***P <* *0.001. See also Supplementary Table 1.

We compared formerly paralysed, recovered and aged P301SxTAU62^*on-off*^ mice to their P301Shet littermates and to non-transgenic BL6 mice by tail suspension, rotarod, and grid climbing at the age of 16 months. P301Shet littermates showed signs of hindlimb clasping, while recovered P301SxTAU62^*on-off*^ mice were still able to spread their hindlimbs, as were non-transgenic BL6 mice (Fig. 3A).

As expected, at 16 months of age, motor balance (rotarod) was significantly reduced in both tau expressing mouse models, compared to non-transgenic BL6 mice. Somewhat surprisingly, however, we did not detect a worse motor performance of P301SxTAU62^*on-off*^ mice in comparison to their P301Shet littermates. In contrast, formerly paralysed P301SxTAU62^*on-off*^ mice performed even slightly better in the rotarod test when compared to P301Shet littermates, although this did not reach statistical significance (Fig. 3B). Similar observations were made for their grid climbing capability, revealing a significantly reduced motor strength in P301Shet transgenic mice in comparison to non-transgenic BL6 mice. Again, at the age of 16 months, P301SxTAU62^*on-off*^ mice showed a slightly better grid climbing performance than their P301Shet littermates, although again without statistical significance (Fig. 3C and Supplementary Table 1). Thus, P301SxTAU62^*on-off*^ mice recovered from early neurotoxic tau stress and did not show increased motor impairment with ageing when compared to P301Shet littermates. These findings are graphically summarized in Supplementary Fig. 2.

### Absence of excessive tau pathology in aged P301SxTAU62^*on-off*^ mice after recovery from tau toxicity

**Figure 4:**
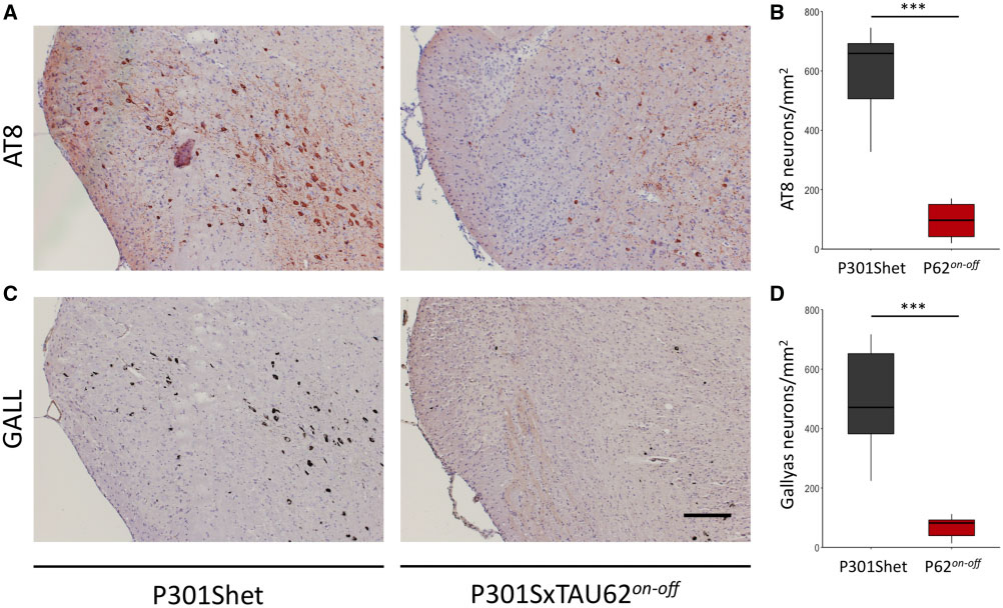
**Aged P301SxTAU62^*on-off*^ mice show decreased hyperphosphorylated and fibrillar tau pathology compared to their heterozygous littermates.** Histological tests comparing AT8 stained brainstem sections (**A**) of 16-month-old P301Shet (*n *=* *7) and P301SxTAU62^*on-off*^ (abbreviated P62^*on-off*^) mice (*n *=* *7) revealed that the different performance in the behavioural tests was paralleled by different degrees of hyperphoshorylated tau pathology (**B**, *P *=* *3.39 × 10^−6^). These results were also mirrored by Gallyas-stained brainstem sections (**C**) of 16-month-old P301Shet (*n *=* *7), and P301SxTAU62^*on-off*^ mice (*n *=* *7), which revealed different degrees of fibrillar tau pathology between the two groups (**D**, *P *=* *7.77 × 10^−5^). ***P <* *0.001. Scale bar = 200 µm (**A** and **C**). See also Supplementary Table 2.

We next aimed to studyassess whether the absence of a pronounced motor phenotype in P301SxTAU62^*on-off*^ mice would also be mirrored by the extent of tau pathology upon ageing. To this end, we comparatively analysed brains of 16-month-old P301SxTAU62^*on-off*^ and P301Shet mice by histology. In all mice, tau pathology was most extensive in the brainstem. AT8 immunohistochemistry revealed extensive tau hyperphosphorylation in P301Shet mice whereas significantly less tau hyperphosphorylation was present in P301SxTAU62^*on-off*^ animals (Fig. 4A and B, for high magnification pictures see Supplementary Fig. 3). Additional immunohistochemistry with the AT100 antibody revealed similar results for 16-month-old mice, confirming the significantly more abundant hyperphosphorylation in the heterozygous mice, which did not experience the early neurotoxic stress as the recovered mice did (Supplementary Fig. 4).

Gallyas-Braak silver staining revealed widespread, robust tau tangle formation in the brainstem of P301Shet mice, whereas formerly paralysed P301SxTAU62^*on-off*^ mice remained almost devoid of fibrillar tau pathology (Fig. 4C and D, for high magnification pictures see Supplementary Fig. 3). Semiquantitative assessment confirmed the lower extent of tau pathology in aged P301SxTAU62^*on-off*^ compared to P301Shet mice (Supplementary Table 2). Immunohistochemistry with TauC3 antibody did not reveal leakage of Δtau_151-421_ expression in 16-month-old P301SxTAU62^*on-off*^ mice (Supplementary Fig. 4). A graphical representation of these findings is shown in Supplementary Fig. 2.

**Figure 5:**
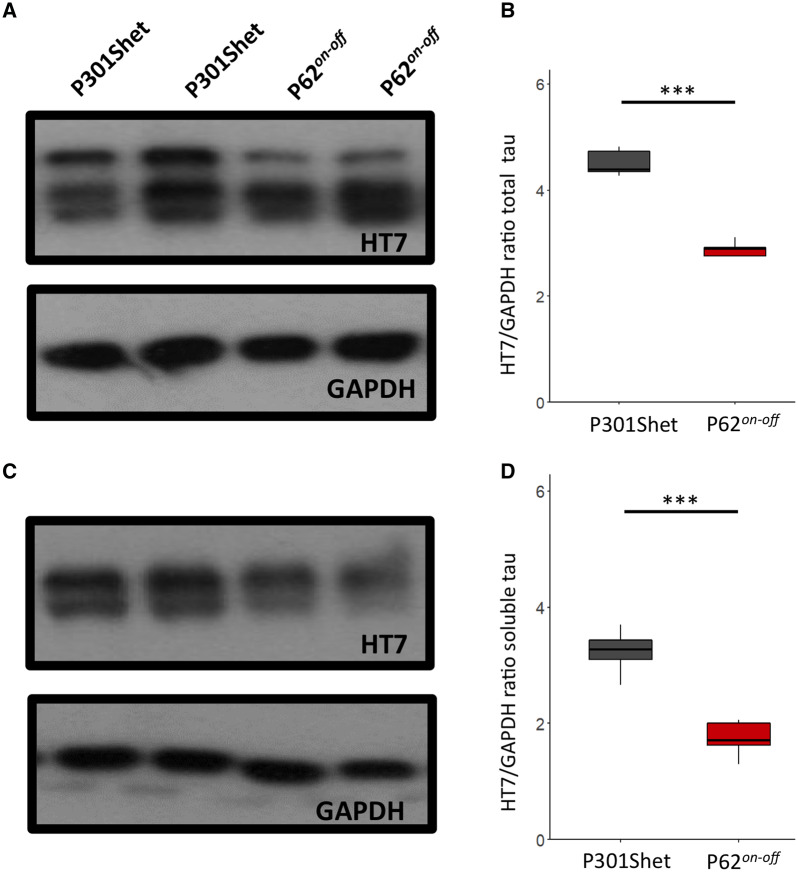
**Aged P301SxTAU62^*on-off*^ mice show lower total and soluble tau levels than their P301Shet littermates.** Western blot analysis of total tau (HT7 antibody) revealed that P301Shet mice (*n *=* *5) have a significantly higher level of total tau when compared to P301SxTAU62^*on-off*^ (abbreviated P62^*on-off*^) mice (*n *=* *5) at 16 months of age (**A**), which was confirmed by HT7/GAPDH ratio quantification (**B**, *P *=* *1.32 × 10^−6^). Soluble tau levels (**C**) in P301Shet mice (*n *=* *5) were also significantly higher than in P301SxTAU62^*on-off*^ mice (*n *=* *5), as reflected by HT7/GAPDH ratio quantification (**D**, *P *=* *0.0001). ***P <* *0.001. See also Supplementary Table 2.

**Figure 6:**
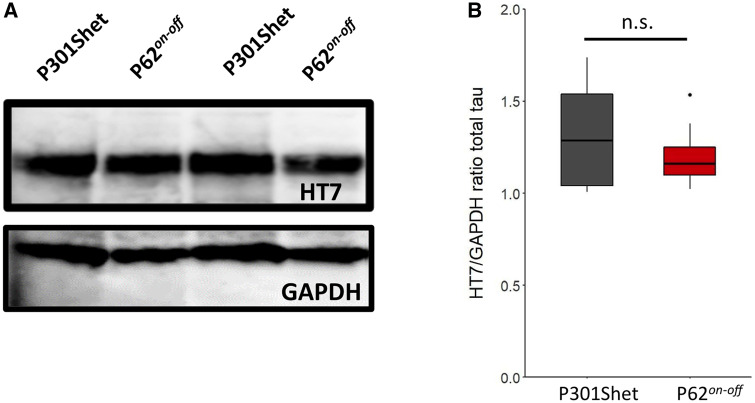
**Total tau levels in young P301SxTAU62^*on-off*^ mice do not decrease compared to their heterozygous littermates.** Western blot analysis (HT7; **A**) and HT7/GAPDH ratio (**B**) for both P301Shet (*n *=* *6) and P301SxTAU62^*on-off*^ (abbreviated P62^*on-off*^) mice (*n *=* *6). Tau levels were not significantly different between the two groups (*P *=* *0.35). n.s. = *P *>* *0.05. Box plots with hinges, whiskers and outliers. See also Supplementary Table 2.

We confirmed our histological findings by western blotting, using the human tau targeting HT7 antibody. Significantly higher levels of total (Fig. 5A and B) and soluble tau (Fig. 5C and D) were detected in P301Shet mice compared to P301SxTAU62^*on-off*^ mice at 16 months of age (Supplementary Table 2). Next, we wanted to rule out that the mild motor phenotype as well as the comparatively mild tau pathology in aged P301SxTAU62^*on-off*^ mice were caused by loss of human mutant P301S tau expression. To this end, we analysed tau protein levels in these mice and their heterozygous littermates at the age of 3 months. Western blot analysis revealed comparable total tau levels in P301Shet and P301SxTAU62^*on-off*^ mice (Fig. 6A and B; see Supplementary Table 2 for complete values). It is therefore unlikely that the maintained motor competence of aged P301SxTAU62^*on-off*^ mice results from a previous major loss of tau-expressing neurons in young paralysed P301SxTAU62^*on*^ mice. Nevertheless, based on this western blotting, discretely reduced tau expression levels in P301SxTAU62^*on-off*^ compared to P301Shet mice cannot be excluded. Therefore, a mildly lowered tau expression may have contributed to the slightly better motor performance and the less extensive tau pathology in aged P301SxTAU62^*on-off*^ mice. In light of the extensive early tau toxicity occurring in P301SxTAU62^*on*^ mice, an immunological reaction could also have been triggered by the toxic high molecular weight tau, and may have caused the lower tau levels in aged P301SxTAU62^*on-off*^ mice. However, dot blot analysis did not detect anti-tau antibodies in sera of 3-month-old P301Shet and P301SxTAU62^*on-off*^ mice (Supplementary Fig. 5).

### Brainstem homogenates from paralysed P301SxTAU62^*on*^ mice do not seed neurofibrillary tangles

**Figure 7:**
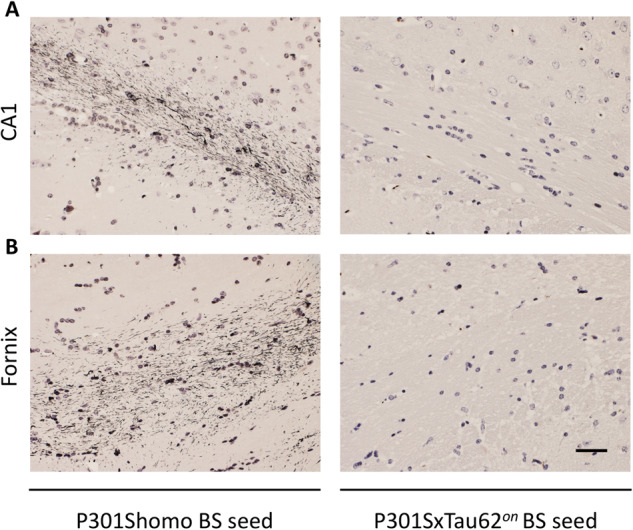
**Seeding with high molecular weight tau results in the absence of fibrils in ALZ17 mice.** ALZ17 mice seeded with brainstem (BS) homogenates from paralysed P301S homozygous (P301Shomo) mice (*n *=* *5) revealed distinct granular tau pathology in CA1 (**A**) and the fornix (**B**), which was not detected in ALZ17 mice seeded with P301SxTAU62^*on*^ brainstem homogenate (*n *=* *4). Scale bar = 37.5 µm (**A** and **B**).

Our observations thus far have argued against a long-term seeding effect of the early, non-fibrillar neurotoxic tau species that cause the severe motor palsy in P301SxTAU62^*on*^ mice. To assess the seeding capacity of these non-fibrillar toxic tau species in a classical tau seeding setting (Clavaguera *et al.*, 2009), we next prepared brainstem homogenates from paralysed 3-week-old P301SxTAU62^*on*^ mice, and inoculated them intrahippocampally into 3-month-old ALZ17 tau transgenic mice. Brain homogenates derived from aged tangle-bearing homozygous P301S tau transgenic mice were used as positive controls, as these had previously been proven to provoke the formation of Gallyas-Braak silver stain-positive tangles when inoculated into ALZ17 mice (Clavaguera *et al.*, 2009). As expected, ALZ17 mice seeded with P301S brainstem homogenates developed marked Gallyas-Braak silver stain-positive granular structures, primarily in CA1 (Fig. 7A) and the ipsilateral dorsal fornix above CA1 (Fig. 7B). In contrast, ALZ17 mice seeded with P301SxTAU62^*on*^ brainstem homogenates remained devoid of such tau pathology up to the age of 20 months. This observation indicates that the non-fibrillar toxic tau species associated with the severe paralysis of P301SxTAU62^*on*^ mice are not seeding-competent. A graphical representation of these findings is provided in Supplementary Fig. 6.

## Discussion

Here we report that early neurotoxic tau stress causing severe motor paralysis in P301SxTAU62^*on*^ mice is fully reversible and does not cause late seeding or adverse effects in recovered P301SxTAU62^*on-off*^ mice during ageing. Furthermore, we found that the inoculation of brainstem homogenates from paralysed P301SxTAU62^*on*^ mice into ALZ17 mice does not result in tau seeding in the host mice, demonstrating that non-fibrillar neurotoxic tau species can lack classical seeding competence.

Co-expression of a Δtau_151-421_ with full-length P301S mutant tau in P301SxTAU62^*on*^ mice causes severe paralysis after 3 weeks, as we have previously reported (Ozcelik *et al.*, 2016). This motor impairment is paralleled by an increase in hyperphosphorylated tau, but occurs in the absence of fibrillar tau forms or tau tangles. Upon switching off Δtau_151-421_ expression, hyperphosphorylated tau species are no longer detectable and P301SxTAU62^*on-off*^ mice regain their motor function. This confirms that hyperphosphorylated non-fibrillar tau oligomers can mediate early neurotoxicity, and that clearance of these tau species rescues the paralysed mice from their motor impairment (Ozcelik *et al.*, 2016).

While overexpression of non-aggregating tau deletion mutants lacks relevant neurotoxicity (Macdonald *et al.*, 2019), we here confirm that oligomeric, yet non-fibrillar tau aggregates can exert extensive tau toxicity. This is also in line with reported clinical data: tau oligomers appear early in the brains of patients developing Alzheimer’s disease (Patterson *et al.*, 2011; Lasagna-Reeves *et al.*, 2012; Koss *et al.*, 2016) and progressive supranuclear palsy (Gerson *et al.*, 2014), possibly even before first clinical symptoms become apparent (Maeda *et al.*, 2006). Tau oligomers have also been shown to correlate well with neurodegeneration in tau transgenic mice (Gomez-Isla *et al.*, 1997; Lasagna-Reeves *et al.*, 2011; Jiang *et al.*, 2019). Based on this, oligomeric tau species are now considered to play a central role in the pathogenesis of neuronal dysfunction in tauopathies (Ghag *et al.*, 2018; Polanco *et al.*, 2018; Sengupta *et al.*, 2018; Bittar *et al.*, 2019), comparable to oligomeric structures in other neurodegenerative disorders (Hong *et al.*, 2018; Sekiya *et al.*, 2019). The severe early neurotoxic stress provoked by non-fibrillar tau forms in our P301SxTAU62^*on-off*^ mice strengthens this view.

Counter-intuitively, here we found that P301SxTAU62^*on-off*^ mice, that have been exposed to non-fibrillar neurotoxic tau, do not show an accelerated course of their tauopathy during ageing. In contrast, aged P301SxTAU62^*on-off*^ mice show better motor performance and less tau pathology in comparison to their heterozygous P301S littermates, even though P301S tau expression remains unaffected after switching off Δtau_151-421_ expression. To our knowledge, this is the first analysis of long-term consequences of early tau stress in a model maintaining the expression of an aggregation-prone tau form. Previous studies were conducted in mouse models conditionally expressing a single mutant human tau form, and the recovery of these mice was achieved by its suppression (Van der Jeugd *et al.*, 2012; Polydoro *et al.*, 2013; Wang *et al.*, 2018). Thus, upon switch-off, aggregation-prone human tau forms were no longer expressed in these mice—a scenario that does not reflect the situation of potential interventional tau clearance in humans. In addition, previous studies in rTgTauEC mice have shown that NFT-associated toxicity can be reversed by suppressing tau overexpression (Polydoro *et al.*, 2013). In pro-aggregant hTau40 transgenic mice, motor fitness improved after stopping mutant tau expression (Van der Jeugd *et al.*, 2012). Similar results were obtained in PS19 mice by antisense oligonucleotide-mediated downregulation of mutant tau expression (DeVos *et al.*, 2013, 2017). Whereas our previous studies showed that tau oligomeric stress can result in severe motor impairment in the absence of tau fibrils (Ozcelik *et al.*, 2016), our present findings further separate tau toxicity from tangle formation by providing the first evidence that early tau toxicity can be reverted, even without subsequent seeding of fibrillar tau tangles. This indicates that halting tau oligomer-related toxicity prior to the appearance of filamentous tau can not only reverse clinical symptoms (as reflected by the motor phenotype recovery of our mice), but may also efficiently prevent long-term tau seeding and spreading. Strikingly, interventions with compounds targeting tau oligomers have already shown beneficial effects, including the reversal of brain dysfunction (Castillo-Carranza *et al.*, 2014; Soeda *et al.*, 2015; Gerson *et al.*, 2018; Lo Cascio *et al.*, 2019; Lo *et al.*, 2019). Our new findings suggest that early therapeutic tau oligomer removal might efficiently prevent the progression of tauopathies.

We cannot exclude that the lack of a late tau seeding effect by early toxic tau species in ageing P301SxTAU62^*on-off*^ mice is caused by immunological reactions targeting tau in these mice. The slower decline of motor function of recovered, ageing P301SxTAU62^*on-off*^ mice could also be associated with a protective mechanism triggered by the early neurotoxic tau stress. While a dot blot analysis of sera of P301SxTAU62^*on-off*^ mice did not reveal antibodies targeting recombinant tau, this does not fully rule out alternative immunological mechanisms (e.g. T cell-mediated or antibodies against specific epitopes of oligomeric tau assemblies). Furthermore, the slightly lowered tau levels in aged P301SxTAU62^*on-off*^ mice could also be a consequence of autophagy induction by the early oligomeric tau stress that these mice experienced; in support of the latter notion, it was previously found that pharmacological autophagy activation can defer tau pathology progression in P301S mice (Schaeffer *et al.*, 2012; Ozcelik *et al.*, 2013).

As we cannot exclude that the lack of a late tau seeding effect by early toxic tau species in P301SxTAU62^*on-off*^ mice is attributable to immunological reactions or the induction of the autophagy pathway targeting oligomeric tau in these mice, we next investigated whether the early toxic tau species exert classical prion-like seeding competence upon intracerebral inoculation. Fibrillar tau induces the aggregation of soluble tau species in a prion-like manner upon inoculation into tau transgenic host mice (Clavaguera *et al.*, 2009; Goedert *et al.*, 2017). We inoculated ALZ17 mice with brainstem homogenates from paralysed P301SxTAU62^*on*^ mice. These seeded ALZ17 mice remained devoid of fibrillar tau pathology up to high ages. In contrast, seedings with P301S brainstem homogenates induced distinct focal granular Gallyas-Braak silver stain-positive tau aggregates, as these homogenates also contain short tau filaments, known to be the most seeding-competent tau species (Jackson *et al.*, 2016). The absence of fibril induction in ALZ17 mice inoculated with brainstem homogenates from paralysed P301SxTAU62^*on*^ mice confirms that early toxic tau oligomers can lack fibrillar tau seeding competence.

At late disease stages, seeding-competent tau structures are present in most tauopathies, and capable of seeding fibrillar tau aggregates when injected into ALZ17 mice (Clavaguera *et al.*, 2013). Interestingly, the only homogenates not provoking the formation of Gallyas-positive fibrils in ALZ17 mice, comparable to our P301SxTAU62^*on*^ brainstem homogenates, were extracts collected from patients with argyrophilic grain disease. This has been linked to the histological predominance of pretangles over fully formed tangles in argyrophilic grain disease (Ferrer *et al.*, 2008), similar to the pretangle stage pathology present in our paralysed P301SxTAU62^on^ mice. A low seeding activity of argyrophilic grain disease extracts has recently also been described in an RT-QuIC *in vitro* seeding model (Kraus *et al.*, 2019). In that study, brain extracts from other tauopathies also exerted only low seeding effects, demonstrating the presence of non-, or not-yet seeding-competent tau forms in human tauopathies including progressive supranuclear palsy and corticobasal degeneration (Kraus *et al.*, 2019). A comparably low seeding efficacy of progressive supranuclear palsy and corticobasal degeneration homogenates was found in a cell-based aggregation assay, while seeds of Alzheimer’s disease patients resulted in high tau aggregation (Chung *et al.*, 2019). In this light, our present findings warrant caution when developing aggregation and seeding-based diagnostic assays. Very early tau forms could be toxic, but undetectable by seeding-based diagnostic tools.

In conclusion, here we demonstrate that high molecular weight tau oligomers can provoke a severe, but reversible neurotoxicity. Rescued mice do not develop long-term sequelae, which argues for early therapeutic interventions targeting oligomeric tau forms. Furthermore, we confirm that these early toxic tau species lack classical seeding competence. Therefore, caution should be exercised when using seeding-based assays for the detection of very early preclinical tauopathy manifestations. Our findings also warrant a deepened analysis of early, non-fibrillar toxic tau forms in the future. While the knowledge on filamentous tau strains is rapidly growing thanks to cryo-electron microscopy studies in particular (Fitzpatrick *et al.*, 2017; Falcon *et al.*, 2018*a*, *b*, 2019), the puzzling characteristics of non-, or not-yet fibrillar tau forms remain only incompletely understood.

## Supplementary Material

awaa445_Supplementary_Data

## Abbreviations

NFT: neurofibrillary tangle
