# Supplementary material for: Severe oligomeric tau toxicity can be reversed without long-term sequelae
Source: Brain. 2021 Jan 23;144(3):963–74. doi: 10.1093/brain/awaa445 (PMC8041046; doi:10.1093/brain/awaa445)
Supplement: awaa445_Supplementary_Data [file awaa445_supplementary_data.zip › brain-2020-00935-File009.pdf]

Supplementary Material

Supplementary Figure 1:

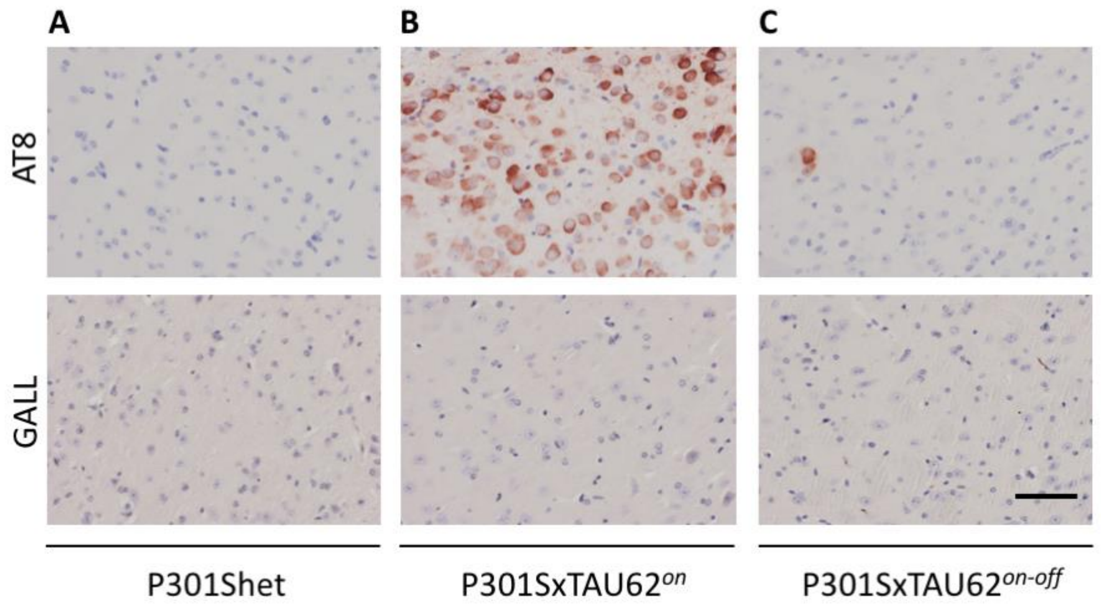

Suppl. Fig. 1: High magnification images extending the findings shown in Figure 1.

P301S heterozygous mice (P301Shet; n=5) at 21 days of age show no signs of hyperphosphorylated tau or Gallyas-positive tau fibrils in the tegmental reticular nucleus (A). Paralyzed P301SxTAU62^{on} mice (n=5) show hyperphosphorylated tau but no Gallyas-positive tau fibrils in the same region (B). After suspending doxycycline administration for 3 weeks, P301SxTAU62$^{on\text{-}off}$ mice (n=5) do not show hyperphosphorylated or fibrillar tau (C). Scale bar equals 50 µm, and applies to A, B and C.

Supplementary Figure 2:

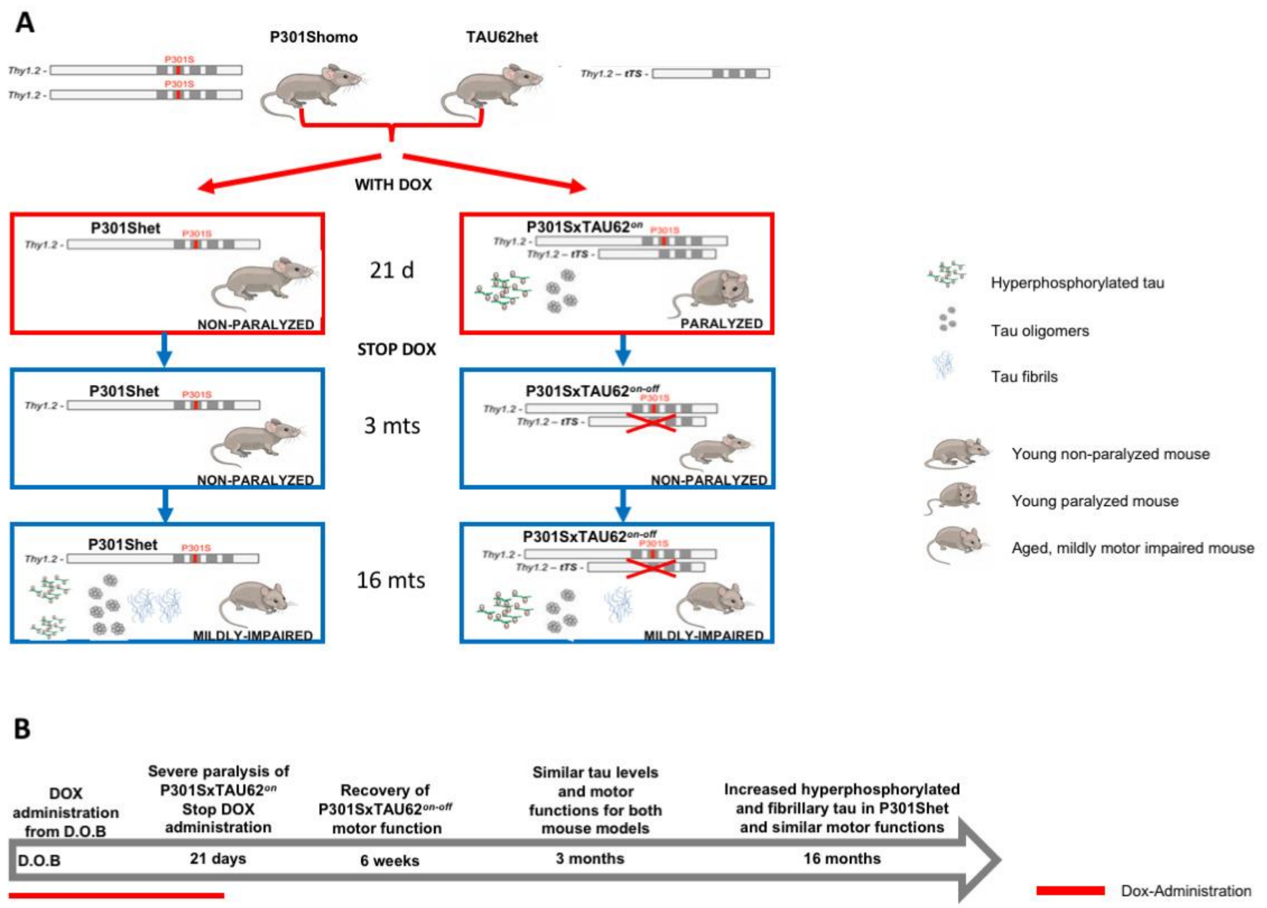

Suppl. Fig. 2: Schematic of background and main conclusions showing no long-term sequelae of reverted tau toxicity.

P301SxTAU62^{on} mice co-express human P301S mutant tau and, only under oral doxycycline (DOX) administration, $\Delta tau_{151\text{-}421}$ fragment. This results in hyperphosphorylated tau oligomers that cause a severe paralysis after 21 days. If these mice are fed normally, they only express heterozygous mutant P301S tau but not the tau fragment, and are thus comparable to P301Shet mice which remain without oligomeric tau formation or severe paralysis after 21 days. After expression of the doxycycline-responsive $\Delta tau_{151\text{-}421}$ fragment is switched off (P301SxTAU62$^{on\text{-}off}$), hyperphosphorylated tau oligomers are cleared efficiently and mice recover their motor function in the following 3 weeks, even though mutant P301S tau expression is being maintained. Therefore, P301SxTAU62$^{on\text{-}off}$ mice exhibit a similar phenotype as P301Shet mice at three months of age. At 16 months of age, P301SxTAU62$^{on\text{-}off}$ mouse motor functions do not differ significantly from P301Shet mice, while tangles and hyperphosphorylated tau oligomers are significantly less abundant in

P301SxTAU62[on-off] compared to P301Shet mice. This proves that P301SxTAU62[on-off] mice do not experience sequelae of the early neurotoxic stress, when compared to their heterozygous P301S tau transgenic littermates.

Supplementary Figure 3:

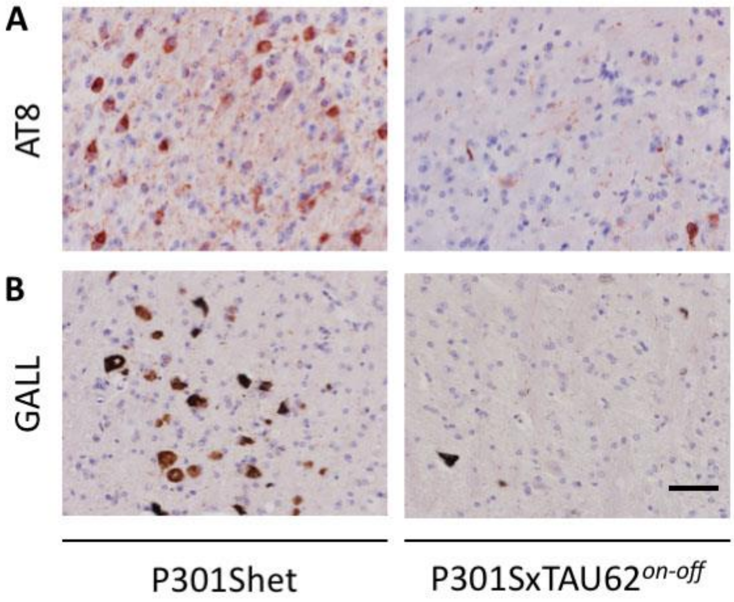

Suppl. Fig. 3: High magnification images extending the findings shown in Figure 4.

Aged P301SxTAU62[on-off] mice exhibit less AT8 and Gallyas-positive neurons than their heterozygous littermates. AT8 (A) and Gallyas silver (B) stained brainstem sections of 16-months-old P301Shet mice (n=7) show more hyperphosphorylated tau and silver stain positive pathology than P301SxTAU62[on-off] mice (n=7). Scale bar equals 50 µm, and applies to A and B.

Supplementary Figure 4:

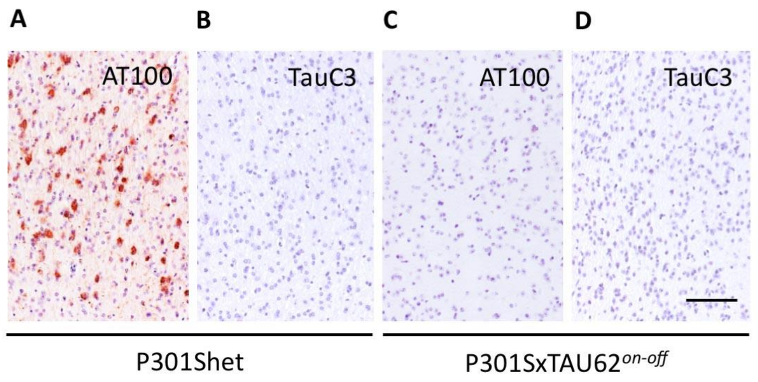

Suppl. Fig. 4: Immunohistochemistry targeting the AT100 phospho-epitopes at Thr 212 and Ser 214, and Δtau (TauC3 antibody) in 16-months-old mice.

In parallel to Gallyas silver stain positivity (Suppl. Fig. 3), extensive hyperphosphorylation of AT100-positive tau was seen in the brainstem of heterozygous P301S littermates (n=7) (A), but was almost absent in P301SxTAU62*on-off* mice (n=7) (C). Immunohistochemistry with TauC3 antibody did not find any tau fragment in non-fragment expressing P301Shet mice (n=7) (B), nor were signs of fragment expression leakage detectable in P301SxTAU62*on-off* mice (n=7) (D). The scale bar corresponds to 100 μm, and applies from A to D.

Supplementary Figure 5:

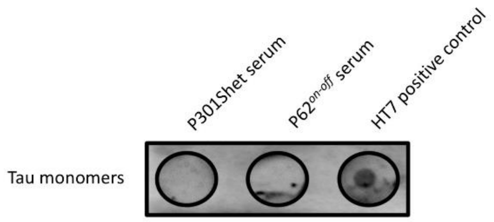

Suppl. Fig. 5: 3-months-old P301Shet and P301SxTAU62*on-off* mice do not show anti-tau antibodies in their sera.

Dot blot with the sera of 3-months-old P301Shet (n=4) and P301SxTAU62*on-off* (labelled P62 *on-off*; n=3) mice was conducted, to test for the presence of anti-tau antibodies, using HT7 antibody as a positive control. 2N4R wild-type tau monomers were applied on the membrane and both sera did not show any immunological activity.

Supplementary Figure 6:

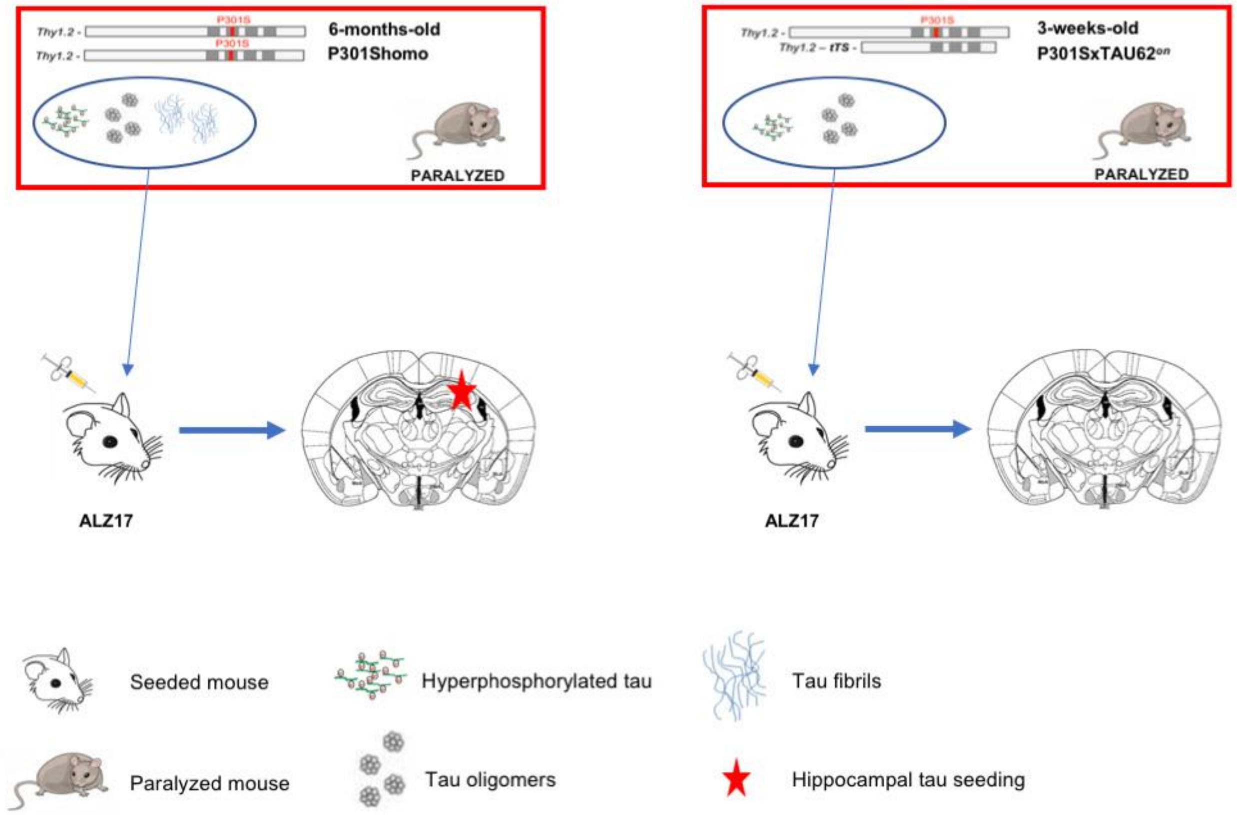

Suppl. Fig. 6: Schematic of background and main conclusions showing that oligomeric toxic tau species can lack seeding competence.

As positive control, ALZ17 mice were injected with tau fibril-containing brainstem homogenates of paralyzed, 6-months-old P301S mice (left box). These ALZ17 control mice developed granular focal tau pathology in the injected hippocampus. Next, we analyzed the seeding capacity of oligomers resulting from co-expression of P301S tau and $\Delta tau_{151-421}$. To this end, we inoculated ALZ17 transgenic mice with brainstem homogenates collected from paralyzed, 3-weeks-old P301SxTAU62^{on} mice, which do not show fibrillary tau pathology (right box). ALZ17 mice seeded with P301SxTAU62^{on} mice brainstem homogenates did not develop focal tau aggregates in the inoculated hippocampus. This proves that brainstem tissue of paralyzed P301SxTAU62^{on} mice lacks *in vivo* tau seeding competence.

Supplementary Table 1:

TEST	***Mean time ± STD BL6***	***Mean time ± STD P62^{on-off}***	***Mean time ± STD P301Shet***	***P-value ANOVA***	***P-value BL6 vs P301Shet***	***P-value BL6 vs P62^{on-off}***	***P-value P301Shet vs P62^{on-off}***
Rotarod 16 months	33 s ± 8,77 s	17,46 s ± 12,78 s	7,73 s ± 12,01 s	2,8E-05 (***)	2,5E-05 (***)	0,01 (*)	0,14 (n.s)
Grid 16 months	81,9 s ± 65,14 s	40,27 s ± 38,84 s	15,35 s ± 17,58 s	0.001 (**)	0,001 (**)	0,4 (n.s)	0.09 (n.s)

Suppl. Table 1: Overview on behavioral tests.

The table shows the overview of the statistical values of the behavioral tests executed on P301S heterozygous mice (P301Shet), P301SxTAU62^{on-off} mice (P62^{on-off}) and BL6 mice; n.s = P-value > 0,05; * = P-value < 0,05; ** = P-value < 0,01; *** = P-value < 0,001.

Supplementary Table 2:

TEST	***Mean ± STD P301Shet***	***Mean ± STD P62^{on-off}***	***P-value P301Shet vs P62^{on-off}***
AT8 neurons/mm^2 16 months	590 ± 149	96 ± 63	3,39E-06 (***)
Gallyas neurons/mm^2 16 months	498 ± 191	68 ± 37	7,77E-05 (***)
HT7/GAPDH signal total tau WB 16 months	4,51 ± 0,24	2,88 ± 0,15	1,32E-06 (***)
HT7/GAPDH signal soluble tau WB 16 months	3,23 ± 0,39	1,71 ± 0,31	0,0001 (***)
HT7/GAPDH signal WB 3 months	1,32 ± 0,3	1,20 ± 0,17	0,35 (n.s)

Suppl. Table 2: Overview on immunohistochemistry and western blots.

The table shows the overview of the statistical values of the quantified stained sections and western blots on 3 and 16 months old P301S heterozygous (P301Shet) mice and P301SxTAU62^{on-off} (P62^{on-off}) mice; n.s = P-value > 0,05; * = P-value < 0,05; ** = P-value < 0,01; *** = P-value < 0,001.
