# Supplementary material for: Severe oligomeric tau toxicity can be reversed without long-term sequelae
Source: Brain. 2021 Jan 23;144(3):963–74. doi: 10.1093/brain/awaa445 (PMC8041046; doi:10.1093/brain/awaa445)
Supplement: awaa445_Supplementary_Data [file awaa445_supplementary_data.zip › brain-2020-00935-File010.pdf]

Figure 5A

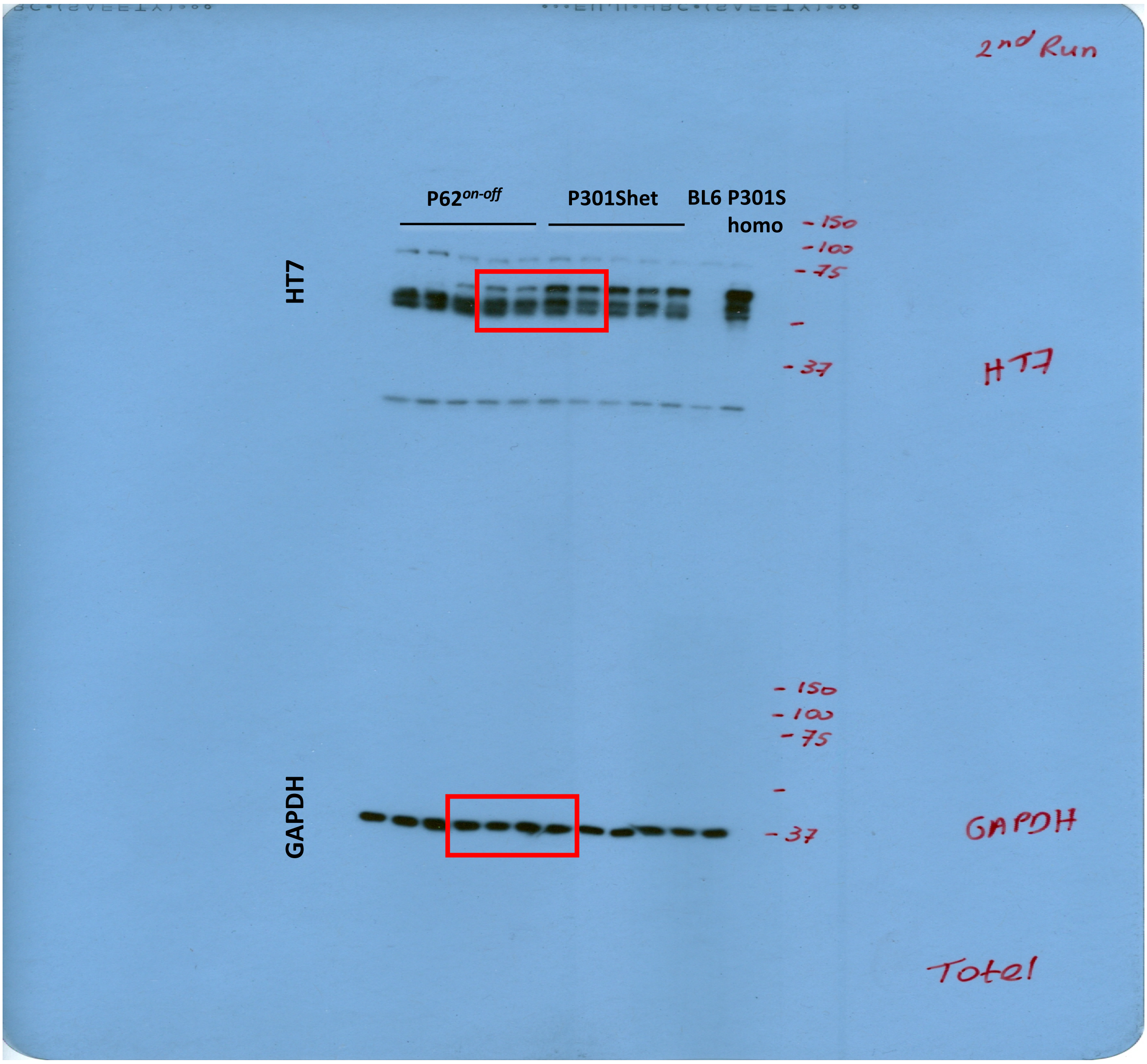

Cropped area (is shown mirrored in the article)

Figure 5C

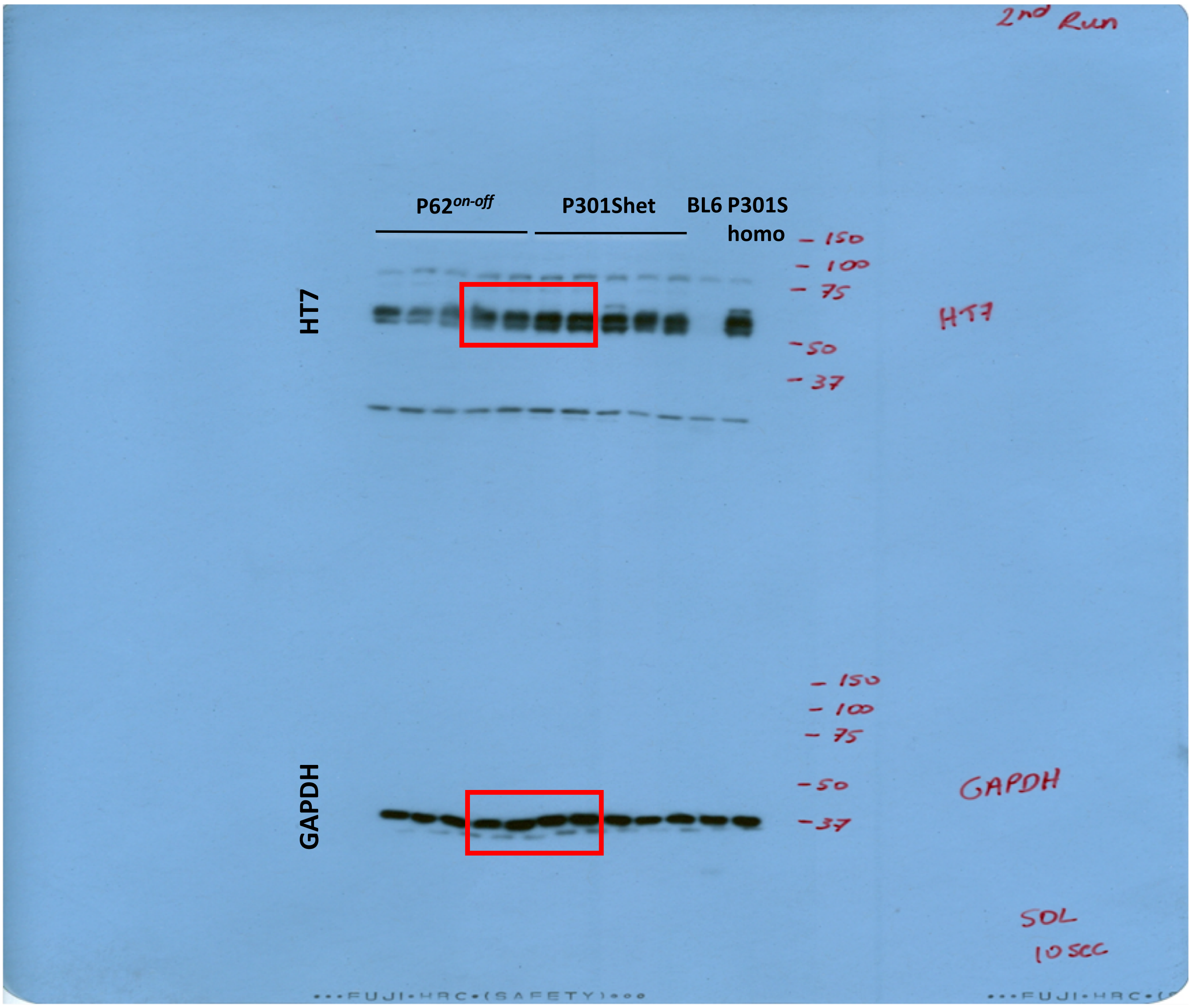

Cropped area (is shown mirrored in the article)

Figure 6A

HT7

GAPDH

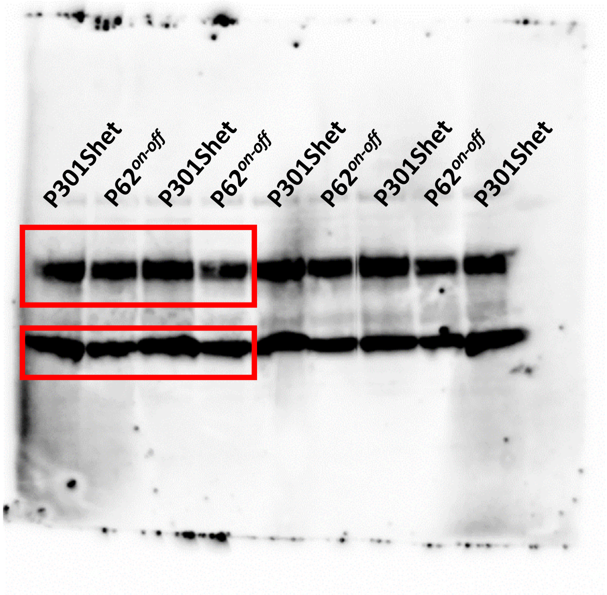

HT7

GAPDH

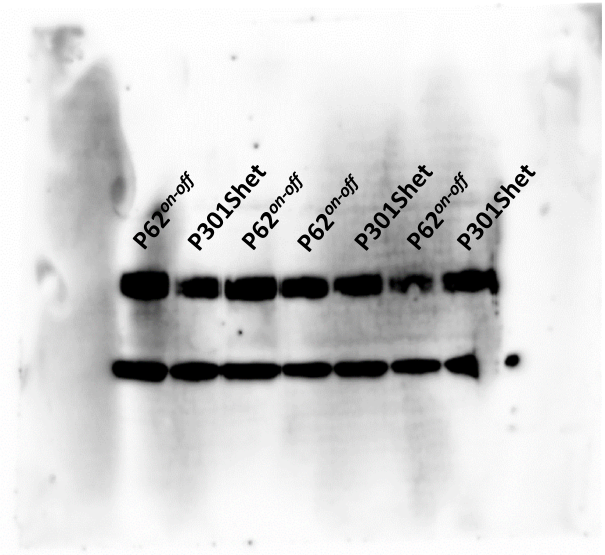

Cropped area
